# High resolution imaging of intracellular oxygen concentration by phosphorescence lifetime

**DOI:** 10.1038/srep10657

**Published:** 2015-06-12

**Authors:** Hiromi Kurokawa, Hidehiro Ito, Mai Inoue, Kenji Tabata, Yoshifumi Sato, Kazuya Yamagata, Shinae Kizaka-Kondoh, Tetsuya Kadonosono, Shigenobu Yano, Masahiro Inoue, Toshiaki Kamachi

**Affiliations:** 1 Department of Bioengineering, Graduated School of Bioscience and Biotechnology, Tokyo Institute of Technology, 2-12-1 Ookayama, Meguro-ku, Tokyo 152-8550, Japan; 2 Education Academy of Computational Life Sciences, Tokyo Institute of Technology, 2-12-1 Ookayama, Meguro-ku, Tokyo 152-8550, Japan; 3 Drug Innovation Research Center, Daiichi University of Pharmacy, 22-1 Tamagawa-cho, Minami-ku, Fukuoka 815-8511, Japan; 4 Department of Medical Biochemistry, Faculty of Life Sciences, Kumamoto University, 1-1-1 Honjo, Chuo-ku, Kumamoto, Kumamoto 860-8556, Japan; 5 Department of Biomolecular Engineering Graduate School of Bioscience and Biotechnology, Tokyo Institute of Technology, 4259 Nagatsuta-cho, Midori-ku, Yokohama, Kanagawa 226-8503, Japan; 6 Graduate School of Materials Science, Nara Institute of Science and Technology, 8916-5 Takayama, Ikoma, Nara 630-0192, Japan; 7 Department of Biochemistry, Osaka Medical Center for Cancer and Cardiovascular Diseases, 1-3-3, Nakamichi, Higashinari-ku, Osaka, Osaka 537-0025, Japan

## Abstract

Optical methods using phosphorescence quenching by oxygen are suitable for sequential monitoring and non-invasive measurements for oxygen concentration (OC) imaging within cells. Phosphorescence intensity measurement is widely used with phosphorescent dyes. These dyes are ubiquitously but heterogeneously distributed inside the whole cell. The distribution of phosphorescent dye is a major disadvantage in phosphorescence intensity measurement. We established OC imaging system for a single cell using phosphorescence lifetime and a laser scanning confocal microscope. This system had improved spatial resolution and reduced the measurement time with the high repetition rate of the laser. By the combination of ubiquitously distributed phosphorescent dye with this lifetime imaging microscope, we can visualize the OC inside the whole cell and spheroid. This system uses reversible phosphorescence quenching by oxygen, so it can measure successive OC changes from normoxia to anoxia. Lower regions of OC inside the cell colocalized with mitochondria. The time-dependent OC change in an insulin-producing cell line MIN6 by the glucose stimulation was successfully visualized. Assessing the detailed distribution and dynamics of OC inside cells achieved by the presented system will be useful to understanding a physiological and pathological oxygen metabolism.

Oxygen is essential for aerobic organisms. Molecular oxygen is required as a terminal electron acceptor in the mitochondrial electron transfer chain for the generation of cellular energy (ATP) and is used as a substrate for numerous enzymatic reactions^1^,^2^. Therefore, oxygen homeostasis is important for maintenance of the cell, tissue, and whole organism. The oxygen concentration (OC) dynamically changes due to an imbalance in consumption and supply in response to the microenvironment and cellular activity. For example, the OC in tumour cells is lower than in normal cells because of poor oxygen supply due to an impaired vascular network. Under hypoxic conditions, hypoxia inducible factor 1 (HIF-1) is induced, and transcriptionally activates the expression of specific genes, including PDK1, which suppresses oxygen consumption. Pancreatic β cells increase oxygen consumption for insulin secretion^3^, and β cells in diabetic mice, in which large amounts of insulin are produced, have been suggested to be hypoxic^4^. Brown adipose tissue also increases oxygen consumption, producing heat via norepinephrine and with subsequent production of uncouplers^5^. Thus, assessing the intracellular OC is fundamentally important to understanding cellular oxygen dynamics.

Experimental approaches to studying tissue and cellular OCs include microelectrodes^6^, electron paramagnetic resonance (EPR)^7^, nitroimidazole adduct staining^8^, and optical methods^9^. Some of the most promising methods for OC imaging within cells and tissues are optical methods, such as two-photon imaging and fluorescence resonance energy transfer (FRET) using luminescence quenching by oxygen^10^,^11^. For example, luminescent dyes, such as polycyclic aromatic hydrocarbons or metallo-complexes (porphyrin compounds or ruthenium complexes, iridium complexes, etc.), are used as oxygen sensing molecules in cells. When a phosphorescent dye, such as platinum (II) tetra (carboxyphenyl) porphyrin (PtTCPP), is irradiated by light, electrons in the ground state are excited to a higher energy level to obtain a photoexcited singlet state, followed by intersystem crossing to form a photoexcited triplet state. Oxygen quenches the photoexcited triplet state; thus, phosphorescence is highly sensitive to the OC^12^. Optical oxygen imaging uses light, so it is suitable for sequential monitoring and non-invasive measurements. Phosphorescence lifetime imaging has two advantages over intensity measurements. First, phosphorescence lifetime measurements do not depend on the concentration of luminescent molecule or exciting light intensity because the decay of the photoexcited molecule ideally obeys first-order kinetics. This is particularly important for measuring OCs inside cells. The distribution of luminescent molecules inside cells is heterogeneous, and the phosphorescence intensity is greatly affected by the concentration of luminescent dye. Second, the effect of background fluorescence can be reduced by measuring the phosphorescence lifetime because fluorescent molecules have a much shorter lifetime than the phosphorescent dye, and the phosphorescence decay can easily be separated from fluorescence decay^13^.

We previously reported an OC imaging system inside a single cell based on phosphorescence lifetime imaging with a microscope^14^. However, the previous optical system did not have enough spatial resolution and required a long time to obtain suitable images. To solve these problems, we developed a new OC imaging system using phosphorescence lifetime measurement with a laser scanning confocal microscope. This system had improved spatial resolution by confocal optical system and accumulation time with the high repetition rate of the laser compared to the original system. Using this system, we investigated the distribution of OC inside a single cell in 2D and 3D culture, as well as alterations in the intracellular OC with changing environmental OC or extracellular stimuli.

## Results

### Phosphorescence lifetime imaging of OC inside a single cell

**Figure 1:**
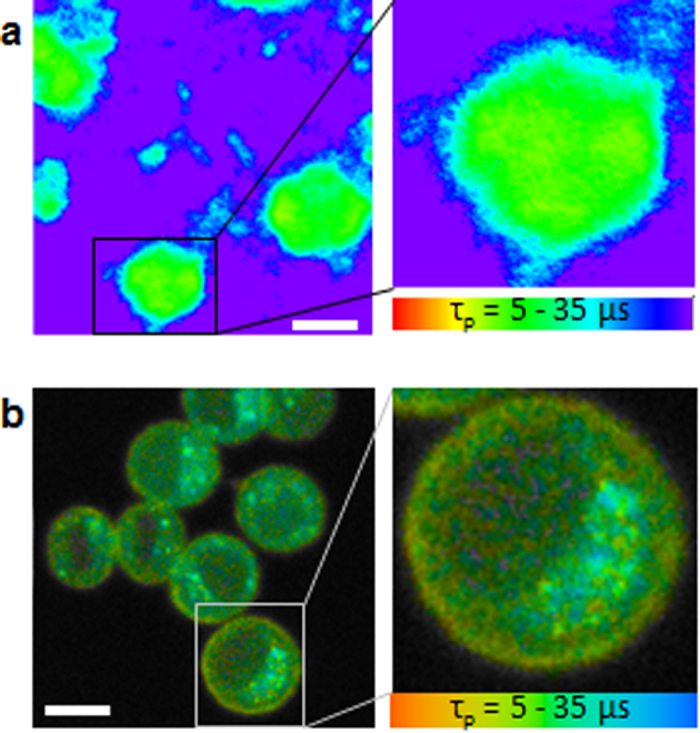
Phosphorescence lifetime imaging of MKN45 cells using a (**a)** fluorescence microscope^14^ and (**b**) confocal microscope. The region colour in red indicates shorter phosphorescence lifetime with a higher oxygen concentration and blue indicates longer phosphorescence lifetime with a lower oxygen concentration. Scale bars = 10 μm.

Figure 1 compares OC imaging in MKN45 cells using the previous wide-field fluorescence microscope^14^ and a confocal microscope (i.e., the present system). A wide-field fluorescence microscope was modified with a gated image intensifier synchronized with a pulse laser to measure the time dependence of phosphorescence intensity decay to estimate the phosphorescence lifetime^14^, which is shown in pseudo-colours. Red colour indicates a shorter phosphorescence lifetime with a higher OC, whereas blue colour indicates a longer phosphorescence lifetime with a lower OC. In the previous wide-field fluorescence system, the obtained phosphorescence lifetime image was so obscure that the fine distribution of OC inside cells was hardly visualized (Fig. 1a). To obtain this image, more than 10 successive phosphorescence intensity images were measured; therefore, it took more than 10 minutes to obtain a phosphorescence lifetime image. In contrast, in the confocal microscope system, the OC inside single cells was clearly visualized (Fig. 1b). The intracellular OC was coloured in green or blue, and the OC in the plasma membrane was coloured yellow, indicating that the OC inside a cell was lower than at the plasma membrane. These results indicate that oxygen is consumed inside the cell. Thus, the combination of a high repetition rate laser (50 MHz) and confocal microscope enables us to obtain higher resolution image of OC inside a single cell, typically within 30–300 s depending on the phosphorescence intensity.

**Figure 2:**
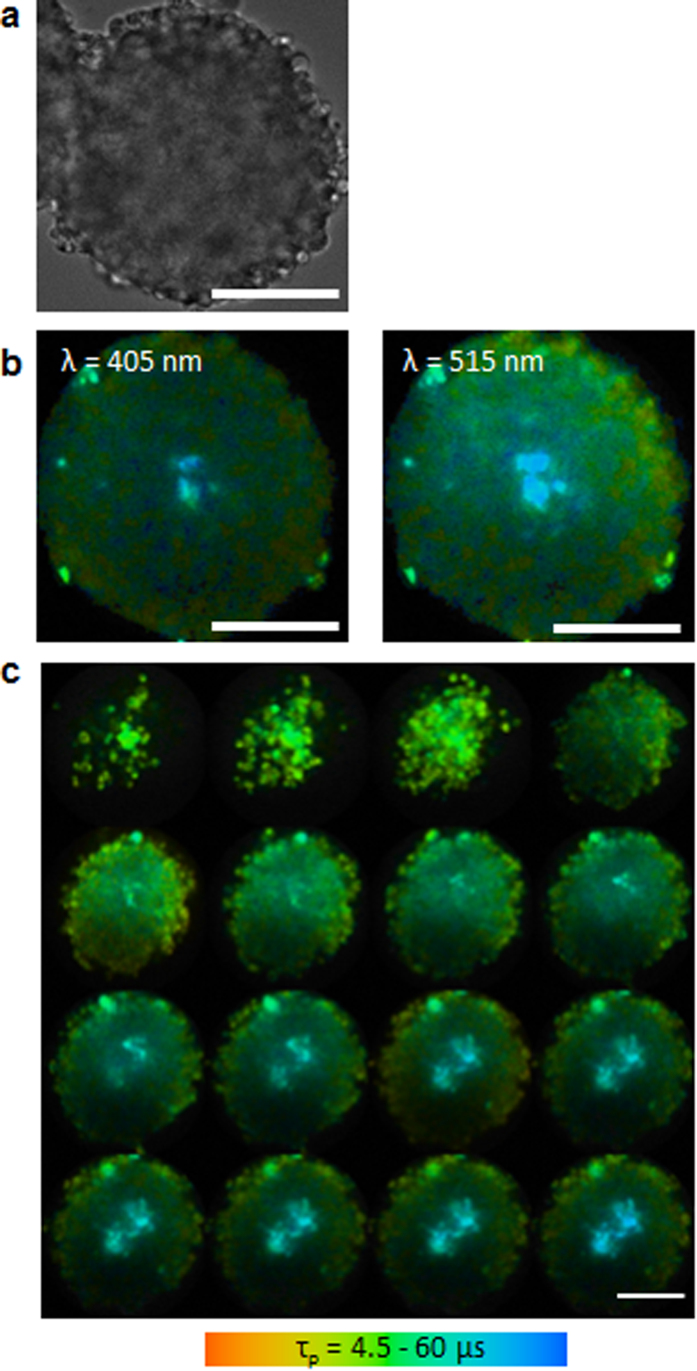
Bright field image (**a**) and phosphorescence lifetime images (**b**, **c**) of a Colon26 spheroid. (**b**) The excitation wavelength was 405 nm (left) and 515 nm (right). (**c**) Z stack images of the Colon26 spheroid are shown from the bottom (upper left image) to upper part (lower right). Each section is at an interval of 10 μm for the Z axis. Scale bars = 100 μm.

The confocal optical system also enables us to obtain cross-section and three-dimensional images of 3D spheroids (Fig. 2). The diameter of the examined spheroid derived from Colon26 cells was ~250 μm (Fig. 2a). Figure 2b shows the effect of laser wavelength on the OC imaging of spheroids. Excitation at 515 nm resulted in better images than 405 nm, especially in the core region of the spheroid. Longer wavelengths of visible light provided better light penetration or transmittance into living tissue, and images obtained with excitation at 515 nm were better in the centre of the spheroid. As phosphorescence lifetime does not depend on the excitation wavelength, no differences were observed in the regional OC pattern of the spheroid with different excitation wavelengths. Figure 2c shows cross-section images of a Colon26 spheroid. Z-series OC images were obtained in a sequence of optical sections collected at 10-μm intervals perpendicular to the optical axis by a piezo actuator. Therefore, the new system is capable of three-dimensional OC imaging.

### Advantages of phosphorescence lifetime imaging for measuring OC inside a spheroid

**Figure 3:**
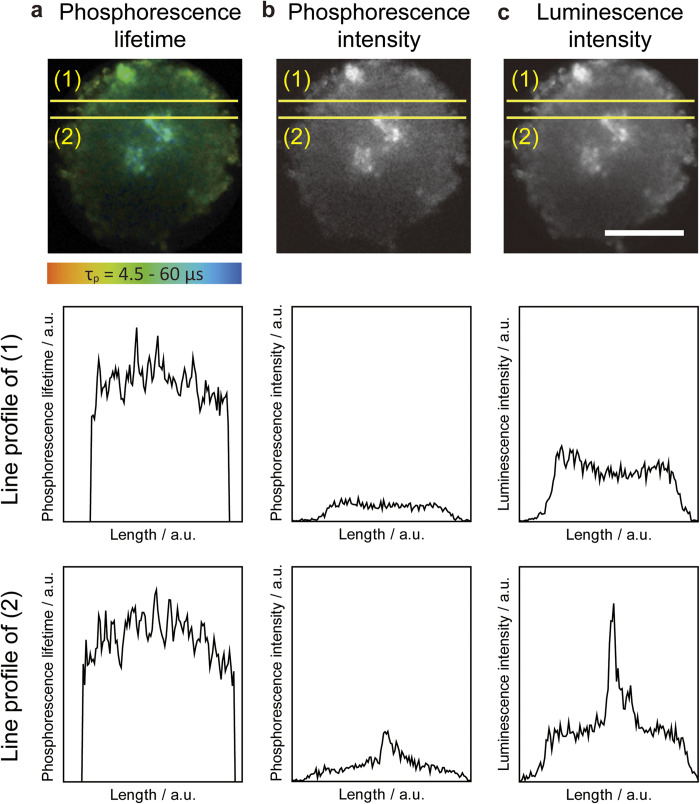
Phosphorescence lifetime (**a**), intensity (**b**), and luminescence intensity (**c**) images of a Colon26 spheroid. The upper panel shows corresponding images of the spheroid. Scale bars = 100 μm. The middle panel shows the line profile of (1) in the upper image and the lower panel shows the line profiles of (2).

As mentioned above, OC can be measured by phosphorescence intensity or lifetime. Phosphorescence intensity has been widely used for OC imaging because it can be measured with a fluorescence microscope, but autofluorescence from cells and phosphorescence from phosphorescent dyes are difficult to separate. The phosphorescence imaging microscope used in this work can measure time-resolved luminescence decay such that fluorescence intensity and phosphorescence intensity can be distinguished. We compared OC images of the Colon26 spheroid obtained by phosphorescence lifetime, phosphorescence intensity, and luminescence intensity (fluorescence plus phosphorescence intensity as observed with a non-time-resolved fluorescence microscope; Fig. 3). The line profiles of the phosphorescence lifetime (Figs. 3a,1 and 2) indicate that the OC of the spheroid was lowest at the core and gradually increased toward the periphery. This result is in agreement with previous reports that the core of the spheroid is hypoxic^15^, which can be explained by oxygen diffusion from outside the spheroid and oxygen consumption by the cells. In contrast, the line profiles of phosphorescence (Figs. 3b,1) and luminescence intensity (Figs. 3c,1) indicate that the high OC area in the core region was surrounded by a low OC area at the periphery of the spheroid. This observation is in contrast to the phosphorescence lifetime image and inconsistent with the fact that the OC inside the spheroid is lower than at the periphery, but it can be explained by the distribution of the phosphorescent dye and the excitation light intensity. Phosphorescent dye diffuses from the periphery to inside the spheroid, and the concentration of phosphorescent dye is higher at the periphery of the spheroid than in the core of the spheroid. In addition, the excitation light intensity at the core of the spheroid can be lower than that at the periphery due to scattering. In the line profile of phosphorescence and luminescence intensity (Figs. 3b,c,2), extremely high intensity regions were observed inside the spheroid. A high intensity region found in the line profile of phosphorescence and luminescence intensity was not observed in the phosphorescence lifetime imaging. These results suggest that phosphorescent dye accumulated in this region and exhibited high phosphorescence or luminescence intensity. As autofluorescence from cells overlapped with phosphorescence, the luminescence intensity was higher than phosphorescence intensity. Thus, information obtained by intensity measurements can be misleading regarding the distribution of OC because the concentration of phosphorescent dye and/or intensity of the excitation light are different in different regions inside a cell or spheroid. On the other hand, phosphorescence lifetime measurement does not depend on the concentration of phosphorescent dye or excitation light intensity. Taken together, the results indicate that phosphorescence lifetime imaging is better than phosphorescence or luminescence intensity imaging for measuring OC inside a cell and spheroid.

### Effect of environmental OC on intracellular OC

**Figure 4:**
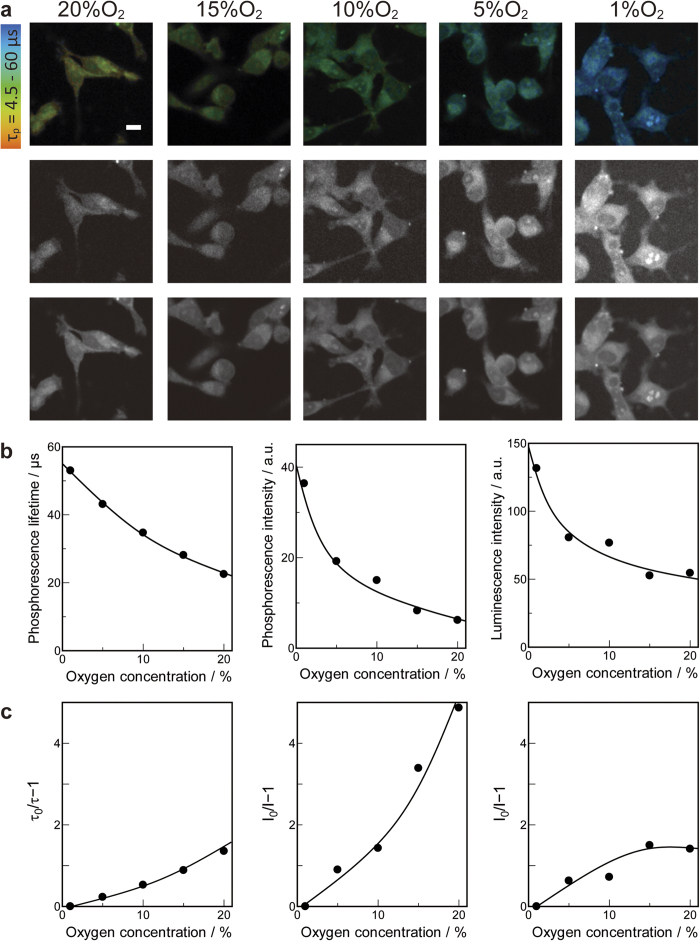
Intracellular oxygen concentration response to changing extracellular oxygen concentration. (**a**) Top: phosphorescence lifetime imaging, middle: phosphorescence intensity imaging, and bottom: luminescence intensity imaging. Scale bars = 10 μm. (**b**) Relationship between oxygen concentration and phosphorescence lifetime (left), phosphorescence intensity (middle), and luminescence intensity (right). Lifetime and intensity represents an average of the data obtained inside cells. (**c**) Stern-Volmer plots. Left: phosphorescence lifetime, middle: phosphorescence intensity, and right: luminescence intensity. Because incubation in 0% oxygen for 1 h caused severe cellular damage, 1% OC was used as τ_p0_, *I*_p0_, and *I*_l0_.

Changes in the intracellular OC in the presence of different environmental OCs was measured using the new system. Colon26 cells in 2D culture were incubated at various OCs for 1 h. After incubation, intracellular OC was measured using a confocal lifetime imaging microscope (Fig. 4). The phosphorescence lifetime inside cells (Fig. 4a, top) became longer by changing the environmental OC from 20% to 1%. The phosphorescence and luminescence intensity inside cells (Fig. 4a, middle and bottom) were also altered by changing the environmental OC. These observations indicate that the phosphorescence lifetime, intensity, and luminescence intensity inside cells depend on the environmental OC. Figure 4b shows the relationship between extracellular OC and the average phosphorescence lifetime, phosphorescence intensity, or luminescence intensity inside cells. All of the measures had a slight downward curvature with environmental OC. Figure 4c shows the plot of phosphorescence lifetime, intensity, and luminescence intensity according to the Stern-Volmer equation (Eq.1):


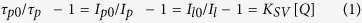


where τ_p0_, *I*_p0_, and *I*_l0_ are phosphorescence lifetime, intensity, and luminescence intensity without oxygen, respectively. Because incubation in 0% oxygen for 1 h caused severe cellular damage, 1% OC was used as τ_p0_, *I*_p0_, and *I*_l0_. *K*_sv_ is the Stern-Volmer constant and [Q] is the environmental OC. If the oxygen consumption inside cells is constant, the Stern-Volmer plot is supposed to be linear, but the phosphorescence lifetime and intensity in Fig. 4c exhibited a downward curve, probably because the intracellular oxygen consumption rate was different at various extracellular OCs so that deviation from the Stern-Volmer equation occurred. Additional deviation observed in the Stern-Volmer plot for luminescence intensity was due to the effect of autofluorescence, which does not depend on the environmental OC.

### Mitochondrial OC

**Figure 5:**
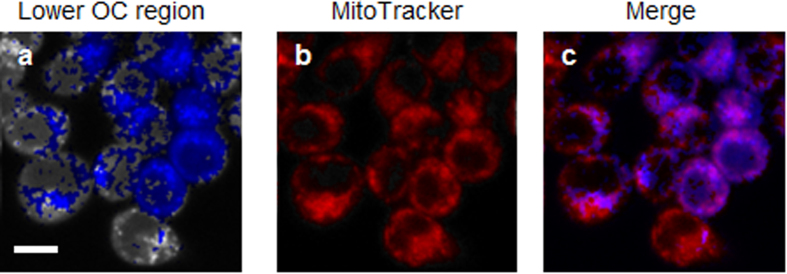
Localization of decreased oxygen concentration in MKN45 cells. (**a**) Lower OC where the phosphorescence lifetime is longer than the average phosphorescence lifetime inside the cell is coloured in blue. (**b**) Labelling of mitochondria with MitoTracker (red). (**c**) Merged images of (**a)** and (**b)**. Purple in the merged image indicates colocalization of the region with lower OC and mitochondria. Scale bar = 10 μm.

The distribution of intracellular OC in MKN45 cells was clearly visualized in Fig. 1b. The OC was heterogeneous inside the cells. We aimed to clarify the characteristics of the lower region of the intracellular OC. As mitochondria generate ATP by consuming oxygen, they are candidates for the region of reduced OC in the cells. Mitochondrion-selective MitoTracker Green FM staining was carried out (Fig. 5). The blue colour indicates the region of lower OC where phosphorescence lifetime is longer than the average phosphorescence lifetime inside a single cell. The MitoTracker stained area colocalized with the region of lower OC. These results suggest that the mitochondrial OC in MKN45 cells is lower than the OC in other components of the cells.

### Effect of inhibited oxygen consumption on intracellular OC

**Figure 6:**
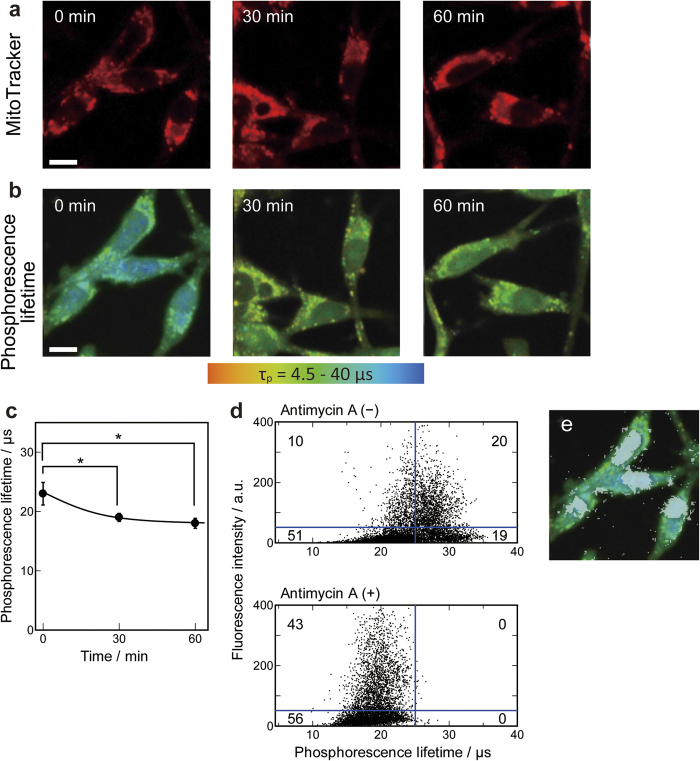
OC in response to the addition of antimycin A to Colon26 cells. (**a**) Labelling of mitochondria with MitoTracker (red). (**b**) Phosphorescence lifetime images. Scale bars = 10 μm. (**c**) Time-dependent change in phosphorescence lifetime after the addition of antimycin A. Data points represent mean ± standard deviations of three independent experiments (*, p < 0.05). (**d**) Dot plot for the relationship between fluorescence intensity of MitoTracker and the phosphorescence lifetime of PtTCPP. Numbers displayed on the figure indicate the population of four distinct sections divided by a quadrant marker. The upper panel is before the addition of antimycin A and the lower panel is after the addition of antimycin A for 60 min. (**e**) Merged image of phosphorescence lifetime (**b**, 0 min) and the lower-right quadrant in the upper panel of (**d**) coloured in white.

To further clarify the role of mitochondrial oxygen consumption in the region of reduced OC, the electron transport chain was inhibited in Colon26 cells. Antimycin A, an inhibitor of complex III in the mitochondrial electron transport chain, is known to reduce the oxygen consumption rate^16^. Figure 6 shows the effect of antimycin A on OC imaging in Colon26 cells. Figure 6a,b show the time-dependent change in OC imaging with MitoTracker staining after the addition of antimycin A. The average phosphorescence lifetime inside cells gradually decreased from 23 μs to 19 μs 30 min after the addition of antimycin A, and this value was sustained at 60 min (Fig. 6b,c). These results revealed that the increase in the OC inside cells by the addition of antimycin A was statistically significant. Figure 6d shows the relationship between the fluorescence intensity of MitoTracker and phosphorescence lifetime of PtTCPP. The 128 × 128 pixel data displayed in Fig. 6d are the data obtained from the phosphorescence lifetime of PtTCPP in Fig. 6b and the fluorescence intensity of MitoTracker in Fig. 6a. The X-axis indicates the phosphorescence lifetime of PtTCPP inside cells; longer phosphorescence lifetime indicates lower OC inside cells. The Y-axis indicates the fluorescence intensity of MitoTracker Green FM; higher fluorescence intensity indicates the existence of mitochondria. The distribution of the plots was divided into quarters. The upper-left quadrant displays a mitochondrion existing in the region with higher OC and the upper-right quadrant displays a mitochondrion existing in the region with lower OC. The lower-left quadrant displays a region with higher OC except for mitochondria, and the lower-right quadrant displays a region with lower OC except for mitochondria. The numbers displayed in the figure indicate the population of four distinguished sections divided by quadrant markers. Before the addition of antimycin A (Fig. 6d, upper panel), approximately two-thirds of mitochondria were found in the lower OC region, whereas all mitochondria were found in the region with higher OC after the addition of antimycin A for 60 min (Fig. 6d, lower panel). The population found in the lower-right quadrant with a lower OC without mitochondrial marker (Fig. 6d, upper panel) was visualized in Fig. 6e as a white region. This result suggests that the population found in the lower-right quadrant in the upper panel of Fig. 6d was located in the cell nucleus. Unlike MKN45 cells, PtTCPP accumulated in the cell nucleus of Colon26 cells and the nuclear OC could be visualized. This region also disappeared after the addition of antimycin A. Therefore, the lower OC in the cell nucleus is suggested to be due, at least in part, to the oxygen consumption by the mitochondria around the cell nucleus. These results suggest that the region of lower OC is caused by the oxygen consumption by mitochondria, and inhibition of the mitochondrial electron transport chain results in the disappearance of the region of lower OC in the cell.

### OC changes in glucose-stimulated MIN6 cells

**Figure 7:**
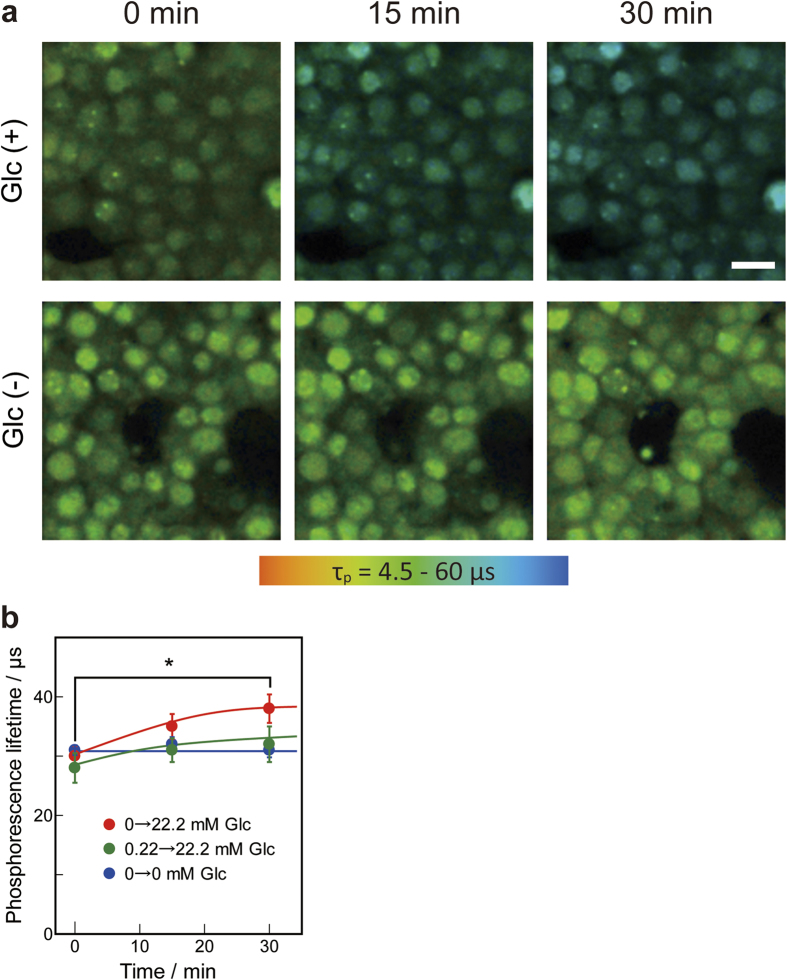
Response of phosphorescence lifetime in MIN6 cells to glucose stimulation. (**a**) At time 0, buffer containing 44.4 mM glucose or buffer without glucose to give a final concentration of 22.2 mM glucose (Glc(+)) or 0 mM glucose (Glc(−)). Scale bars = 10 μm. (**b**) Time-dependent change in phosphorescence lifetime with the addition of glucose. The concentration of glucose increased from 0 to 22.2 mM (red), 0.22 to 22.2 mM (green), and 0 to 0 mM (blue). Data points represent mean ± standard deviations of three independent experiments (*p < 0.05)

MIN6 cells, an insulin-producing cell line derived from murine pancreatic islets^17^, consume large amounts of oxygen for insulin secretion by glucose stimulation^18^. In our previous study, the oxygen consumption rate of MIN6 cells was assessed by measuring the OC of the buffer in a closed chamber over time using a Clark-type oxygen electrode system. The oxygen consumption rate was higher when MIN6 cells were cultured with a higher concentration of glucose^19^. We imaged MIN6 cells to determine the OC change after the addition of glucose under 20% oxygen. As shown in Fig. 7a, the OC in MIN6 cells decreased when the glucose concentration was increased from 0 to 22.2 mM, but the OC did not change without glucose stimulation. In addition, the OC decrease in MIN6 cells was larger when the glucose concentration was increased from 0 to 22.2 mM than from 0.22 to 22.2 mM (Fig. 7b). When the glucose concentration was increased from 0 to 22.2 mM, the OC decrease in MIN6 cells was statistically significantly. These results indicate that intracellular OC decreases with glucose stimulation.

## Discussion

In this study, we report a high resolution imaging system for observing OC inside a cell using phosphorescence lifetime measurement under a confocal microscope. A previous system using a wide-field microscope measured the phosphorescence from all points in the focal plane at once and all light from illuminated regions of the sample that are above and below the focal plane^14^,^20^. Therefore, the previous system was not able to obtain high resolution. Using the new system, we revealed that the OC inside cells is lower than the OC at the plasma membrane. The difference can be explained by oxygen diffusion across the plasma membrane and oxygen consumption inside the cell by respiration. The intracellular OC has been reported to be affected by the oxygen consumption of the electron transfer chain in mitochondria^21^. Dmitri *et al*. reported the assessment and quantitation of pericellular and intracellular molecular oxygen using Pt-porphyrin-based probes and a phosphorescence lifetime-based O_2_ sensing technique; respiring cells exposed to various levels of atmospheric O_2_ exhibited differences in the oxygenation of their pericellular and intracellular compartments^22^. However, that report used nanoparticles for measuring oxygen; they enable better cellular uptake but accumulate in particular parts of the cell, such as lysosomes or the endoplasmic reticulum, so that OC measurement can only be achieved near the particles inside these paticular organelles. As each particles emits the same intensity of phospohrescence, the phophorescence intensity represents the oxygen levels, if phosphorescence intensity from each nanoparticle can be imaged separately. The new system uses phosphorescent dye, which is ubiquitously but heterogenously distributed inside the whole cell. As our system measured OC using phosphorescence lifetime of the phosphorescent dye, the heterogeneous distribution of the phosphorescent dye does not affect the OC measurement; lifetime measurement does not depend on the concentration of the phosphorescent dye. This advantage enables us to visualize the OC in each organelle. By combining our sytstem with organelle-specific staining, the region of lower OC inside the cell was shown to be mitochondria. Furthermore, the OC of the cells inside the multicellular spheroid was lower than the OC in a 2D cultured single cell. The spheroid is much larger than a 2D cultured cell, and the distance from the periphery to the core is much greater than that of a single cell, resulting in much less oxygen diffusion and more oxygen consumption inside the spheroid.

The OC imaging system used in this paper measures phosphorescence lifetime because phosphorescence lifetime is not affected by the concentration of phosphorescent dye and intensity of the excitation light. Chemical compounds generally distribute inhomogenously inside the cell, so phosphorescence and luminescence intensity measurements can provide misleading OC images. These observations indicate that intracellular OC imaging with phosphorescence lifetime is more suitable than measuring phosphorescence intensity.

Phosphorescence quenching by oxygen is suitable for measuring the OC in living cells and tissues. Recently, Spencer *et al*. directly measured oxygen tension in the bone marrow interstitial space *in vivo* and demonstrated that the OC in the interstitial space of the bone marrow is quite low^23^. Our measurement system using a confocal microscope enabled direct observation of the OC inside cultured cancer cells in both 2D and 3D. Imaging the OC inside a cell would provide useful information to elucidate the characteristics of oxygen metabolism in cancer cells and to develop novel therapies.

Pimonidazole is one of the 2-nitroimidazoles and widely used as a hypoxia marker in cells. Under hypoxic conditions, reduction leads to the production of a hydroxylamine intermediate, and adduct formation occurs by irreversibly binding to thiol groups of cysteine residues^24^. Thus, pimonidazole dose not directly measure oxygen tension, but rather redox potential change inside cells, and is not suitable for real-time imaging. In contrast, the system presented here uses reversible phosphorescence quenching by oxygen, so it can measure not only the OC under hypoxic conditions, but also successive OC changes from normoxia to anoxia. The time-dependent change in OC inside single cells can also assessed. As shown in Fig. 4, the effect of a successive decrease in the extracellular OC to the intracellular OC can be measured easily by the new system. From Fig. 4c, the OC or phosphorescence lifetime dependency of the extracellular OC is deviated from the Stern-Volmer equation. The oxygen consumption rate inside the cell may decrease when the OC in the extracellular environment decreases because of a shift to the glycolytic metabolic pathway^25^.

The distribution of intracellular OC was clearly visualized using the new system. Lower regions of OC inside the cell colocalized with mitochondria. This observation was further demonstrated by the complex III inhibitory experiment using antimycin A. The intracellular region of lower OC disappeared with the addition of antimycin A, indicating that low OC inside a cell is caused by the mitochondria. A decrease in the ATP concentration by inhibiting the mitochondrial electron transport probably affects intracellular activity so that the oxygen consumption by other metabolic enzymes also decreases. Interestingly, the nuclear OC was also lower than the OC in the cytoplasm before the addition of antimycin A, whereas the nuclear OC was almost the same as the OC in the cytoplasm after the addition of antimycin A. The lower nuclear OC may be due to the mitochondrial oxygen consumption around the cell nucleus. Oxygen consumption by the cell nucleus can also be responsible for the lower nuclear OC because some of the nuclear enzymes, such as demethylases, including JMJ and TET, use oxygen as a substrate^26^.

The OC in MIN6 cells, an insulin-producing cell line, promptly decreased after glucose stimulation, even under 20% oxygen. The rapid decrease in OC inside cells can be attributed to the oxygen consumption by insulin secretion after glucose stimulation. Our previous study indicated that high glucose concentration yielded a more prominent induction of HIF-1α than low glucose concentration at 10% oxygen. On the other hand, HIF-1α induction was not observed at 20% oxygen, even with high glucose concentrations. Thus, our OC imaging method detected the decrease in OC in MIN6 cells at levels that are too high to induce HIF-1α. Biphasic insulin secretion is the normal response of β cells to a rapid and sustained increase in glucose concentration^27^. Incubation for 15 and 30 min after glucose stimulation corresponds to the second phase of insulin secretion. Second phase insulin secretion is sustained flat or gradually increases depending on the glucose concentration. In this study, intracellular OC decreased gradually (Fig. 7). This change is similar to the gradual increase in the second phase insulin secretion. Thus, the intracellular OC change caused by glucose stimulation may correlate with insulin secretion. ATP consumption during insulin secretion may result in the oxygen consumption for ATP synthesis.

In conclusion, we established an OC imaging system for a single cell using phosphorescence lifetime and a laser scanning confocal microscope. The OC inside of both 2D and 3D cultured cells can be measured by this method. A region of lower OC colocalized with mitochondria. This system also enables us to measure the time-dependent OC after the addition of external stimuli. Assessing the detailed distribution and dynamics of OC inside cells will be useful to understanding physiological and pathological oxygen metabolism.

## Methods

### Cell cultures

The human gastric cancer cell line MKN45 and mouse colon carcinoma cell line Colon26 cells were obtained from Riken cell bank and cultured in RPMI1640 containing 10% FBS, penicillin and streptomycin. Formation of spheroids from Colon26 cells was carried out using Corning Ultra-Low Attachment surface according to the manual with some modifications as follows. Colon26 cells cultured in traditional tissue culture were trypsinized and seeded at a density of 1 × 10^4^ cells/mL in RPMI1640 containing 10% FBS, penicillin, streptomycin and B27 supplement on to Corning Ultra-Low Attachment surface. Mouse insulinoma cell line MIN6 were cultured in DMEM containing 4.5 g/L glucose, 10% FBS, penicillin, streptomycin and 50 μM β-mercaptoethanol. Cells and spheroids were cultured at 37 °C under 5% CO_2_, 95% air conditions. Hypoxic culture was performed in a Stage Top Incubator (Tokai Hit).

### Photosensitizer

Platinum(II) 5,10,15,20-tetrakis-(4-carboxyphenyl)porphyrin (PtTCPP) was synthesized in our laboratory from TCPP^28^,^29^. PtTCPP was dissolved in DMSO and used as stock solution.

### PtTCPP uptake

Colon26 cells were seeded in 35 mm glass based dish at a density of 2 × 10^5^ cells per 2 mL of medium. MKN45 cells were seeded in 35 mm glass based dish at a density of 4 × 10^5^ cells per 2 mL of medium. After overnight incubation, these cells were rinsed with PBS twice and incubated in phenol red-free RPMI1640 containing 10 μM PtTCPP in the dark for 1 h at 37 °C, 5% CO_2_. After incubation, cells were rinsed with PBS twice and added fresh phenol red-free RPMI1640 and were applied for OC imaging.

Spheroids were incubated in phenol red-free RPMI1640 containing 10 μM PtTCPP in the dark for 24 h at 37 °C, 5% CO_2_. After incubation, spheroids were put onto 35 mm glass based dish in 2 mL phenol red-free RPMI1640 and were applied for OC imaging.

MIN6 cells were seeded in glass based chamber at a density of 5 × 10^4^ cells per 250 μL of medium. After incubation for few days, cells were rinsed with PBS twice and incubated in phenol red-free RPMI1640 containing 10 μM PtTCPP in the dark for 6 h at 37 °C, 5% CO_2_. Cells were rinsed with PBS twice and added 250 μL Krebs-Ringer-bicarbonate HEPES (KRBH) buffer and were applied for OC imaging.

### Phosphorescence lifetime and intensity measurement

Phosphorescence lifetime imaging were performed on a fluorescence microscope (Nikon, TE2000-U) equipped with confocal laser scanning system (DCS-120, Becker&Hickl). PtTCPP was excited at 405 or 515 nm pulsed diode laser (pulse duration 60 ps, range of pulse repetition rate 50 MHz, Becker&Hickl) and detected through a 435 or 525 nm long path filter. Cells were maintained at 37 °C, 5% CO_2_ and humidified in a Stage Top Incubator during measurement of OC imaging. Phosphorescence emitted from PtTCPP in the cell was detected by the hybrid photomultiplier tube (HPM-100-40, Becker&Hickl) by through an oil objective lens (Plan Apo TIRF 60 × 1.45 NA , Nikon) and measured time-correlated single photon counting system (SPC-150, Becker&Hickl). The obtained phosphorescence decay curve in each pixel was fitted with a single exponential function using SPCImage software (Becker&Hickl).

### Mitochondria stain

MKN45 cells were rinsed with PBS twice and incubated in phenol red-free RPMI1640 containing 1 μM MitoTracker Green FM (Invitrogen) in the dark for 30 min at 37 °C, 5% CO_2_ after PtTCPP uptake. After incubation, cells were rinsed with PBS twice and added 2 mL fresh phenol red-free RPMI1640 and were applied for OC and mitochondrial imaging. MitoTracker was excited at 515 nm and the emission was collected at 525/50 nm bandpass filter.

### Inhibition of mitochondrial complex III

After accumulation of PtTCPP and MitoTracker as mentioned above, Colon26 cells were added 1 mL phenol red-free RPMI1640 and incubated at 37 °C, 5% CO_2_ and humidified in a Stage Top Incubator for 30 min before addition of antimycin A. Antimycin A solution was added to cell culture medium through perfusion block, which is fitted in the Stage Top Incubator. The temperature or OC of culture medium did not change by the addition of antimycin A solution. OC imaging was performed before and after addition of 4 μM antimycin A. Final concentration of antimycin A in medium was 2 μM. The experiments were repeated three times.

### Glucose stimulation

After accumulation of PtTCPP as mentioned above, MIN6 cells were incubated for 30 min in 250 μL KRBH buffer with 0.22 mM glucose or without glucose in the Stage Top incubator. After incubation, cells were added 250 μL KRBH buffer with glucose or without glucose through perfusion block. The temperature or OC of culture medium did not change by the addition of glucose solution. When cells were added KRBH buffer with glucose, final concentration of glucose in buffer was 22.2 mM. The experiments were repeated three times.

### Statistical Analysis

Unpaired t-test statistical analysis was carried out for the phosphorescent lifetime. Data were compared before and after addition of antimycin A or glucose. A value of p < 0.05 was considered to be statistically significant.

## Additional Information

**How to cite this article**: Kurokawa, H. *et al*. High resolution imaging of intracellular oxygen concentration by phosphorescence lifetime. *Sci. Rep*. **5**, 10657; doi: 10.1038/srep10657 (2015).
